# Predicting of survival in metastatic melanoma patients under anti-PD-1 monotherapy using genomic mutation and copy number variation

**DOI:** 10.1007/s12672-026-05221-8

**Published:** 2026-05-18

**Authors:** Yu Wang, Wei Tang, Yuanyuan Wang, Xiaoyu Lu, Ling Zhang, Jiaojiao Yang, Shuibing Yang, Jingjin Yang

**Affiliations:** 1 Department of Endocrine Metabolism and Clinical Nutrition, Hunan University of Medicine General Hospital, Jinxi South Road, Huaihua, 418000 Hunan China; 2 The College of Public Health, Shanghai University of Medicine & Health Sciences, 279 Zhouzhu Road, Pudong New Area, 201318 Shanghai, China; 3 Jinshan District Central Hospital Affiliated to Shanghai University of Medicine & Health Sciences, 201599 Shanghai, China

**Keywords:** Anti-PD-1 monotherapy, Metastatic melanoma, Survival identification, Predictive model, Genomic data

## Abstract

**Background:**

Although PD-1 inhibitors have significantly reduced metastatic melanoma patients’ mortality, only a small proportion of these patients actually benefit. We aim to develop some parsimonious predictive models for identifying those patients’ survival outcome under anti-PD-1 treatment.

**Method:**

Based on the genomic mutation and copy number variation data of metastatic melanoma patients in the Liu and Shoushtari cohorts, we developed and validated three different nomograms to identify their survival outcomes under anti-PD-1 monotherapy. Patients without anti-PD-1 monotherapy in the TCGA and MSK cohorts were employed as negative controls to assess the applicability of our models to anti-PD-1 monotherapy. All predictive models developed in this study are accessible and available for use at https://yyw0505.shinyapps.io/nomogram_app/.

**Result:**

The first nomogram, and also the one we most recommend, built on five genes including NID1 amplification, TYRP1 deletion, mutations of PIK3C2G, FLT1 and IKZF1, classified patients into the High-Risk and Low-Risk group, and patients in the High-Risk group exhibited shorter overall survival (OS) and progression-free survival (PFS). However, there was no difference of OS between the two groups in both the TCGA and MSK cohorts, indicated the first nomogram was a predictive model rather than prognostic model. Similar results were also observed in the second and third nomogram.

**Conclusion:**

The parsimonious and robust predictive model could provide valuable assistance for clinical practice.

**Supplementary Information:**

The online version contains supplementary material available at 10.1007/s12672-026-05221-8.

## Introduction

Melanoma has the highest mortality among all skin cancers, and the survival rates of patients decline drastically once the cancer metastasized [1–3]. In recent years, although the use of immune checkpoint inhibitors, such as PD-1 inhibitors, has significantly reduced the mortality in patients with metastatic melanoma, only a relatively small proportion of patients experience true clinical benefits from anti-PD-1 therapy. There are several biomarkers employed to predict the response of metastatic melanoma patients to PD-1 inhibitors, such as the expression of PD-L1 measured by immunohistochemistry in tumor cells [4–8], tumor mutational burden (TMB) and neoantigen load [9–11], and so on. However, the expression of PD-L1 lacks a standardized cutoff value and is relatively expensive [12]. TMB is a measure of the number of genomic mutations in a tumor, and the United States Food and Drug Administration (FDA) has approved that TMB ≥ 10 mutations/megabase as a biomarkers of response to immunotherapy in solid tumor [13]. However, Strickler et al. [14] reported that TMB as a predictor of immunotherapy response varied significantly depending on tumor histology and a universal TMB threshold might not be appropriate.

Currently, several studies are dedicated to predicting the response to PD-1 inhibitors in advanced melanoma patients, with the response assessed according to RECIST criteria v1.1 [15]. Zhang et al. [16] first defined responders as patients exhibiting complete response (CR), partial response (PR), or stable disease (SD) with progression-free survival (PFS) ≥ 6 months. Then, they identified responders in melanoma patients with anti-PD-1 therapy or combination immunotherapy based on their genomic mutation and copy number variation (CNV) data. Zila et al. [17] identified responders (CR, PR or SD) from advanced melanoma patients who receiving at least PD-1 inhibitor therapy using their proteomic profiles. However, in the both studies above, melanoma patients were likely to receive other immunotherapy in addition to PD-1 inhibitors, and the outcomes of immune therapy were assessed using surrogate endpoints.

In this study, we have developed three different nomograms based on genomics mutation and CNV profiles of metastatic melanoma patients who received anti-PD-1 monotherapy. These proposed models utilize a comprehensive molecular signature to identify the survival outcomes of metastatic melanoma patients under anti-PD-1 monotherapy, addressing most of the limitations associated with current biomarkers.

## Materials and methods

### Clinical cohorts

In this study, we utilized four cohorts comprising patients with metastatic melanoma and each patient had a genomic mutation profile and CNV profile. The Shoushtari cohort, derived from Memorial Sloan Kettering Cancer Center (MSKCC) dataset, consisted 153 metastatic melanoma patients who had clinical information, pre-treatment genomic mutation profile and CNV profile, and were treated with anti-PD-1 monotherapy. These patients were molecularly profiled by MSK-IMPACT Sequencing, and all of them had tumor purity > 20%. A comprehensive explanation of the gene sequencing technology and clinical information can be found in Shoushtari et al. [18]. Within the Shoushtari cohort, there were 122 (79.74%) patients diagnosed with cutaneous melanoma (Supplemental Fig. 1).

A total of 144 metastatic melanoma patients, who were profiled by whole exome sequencing before treated with anti-PD-1 monotherapy, were identified from the study conducted by Liu et al. [19]. After excluding one patient with post-treatment tumor tissue and ten patients with tumor purity < 20%, the Liu cohort consisted of 97 cutaneous melanoma patients and 36 patients with other types of melanomas (Supplemental Fig. 1).

In addition, we screened 348 patients with metastatic melanoma from The Cancer Genome Atlas (TCGA) dataset, who with tumor purity ≥ 20% and had not treated with PD-1 inhibitors, to form the TCGA cohort. Similarly, we included 214 metastatic melanoma patients from the MSKCC dataset who with tumor purity ≥ 20% and without immunotherapy to form the MSK cohort.

In this study, we strictly focused on non-silent genomic mutations, assigning a value of 0 to the wild-type gene and 1 to the mutant gene. According to research by Bai and Zhang [20, 21], the rarely mutated genes might cause bias for the predictive model, we excluded genes with a mutation frequency of less than 5%. The CNV were classified as amplification or deletion based on $\:\left|{\mathrm{log}}_{2}\left(copy\:ratio\right)\right|>0.5$. In the amplification profile, a value of 1 was assigned to a gene if it was amplified, while a value of 0 was assigned if it was not amplified. The genes with variation rate < 5% were excluded from the CNV amplification profile. Similarly, in the gene deletion profile, a value of 1 was assigned if a gene was deleted, and 0 was assigned conversely. Given the low rate of CNV deletion variation, we only excluded genes with variation rate < 2% in the CNV deletion profile.

## Study design

The primary objective of this study is to predict the survival outcomes of patients with metastatic melanoma undergoing anti-PD-1 monotherapy using their genomic mutation and CNV data. Among the four cohorts, the Liu cohort had survival outcomes assessed in terms of overall survival (OS) and PFS, while the remaining three cohorts had OS as the measure of survival. The OS was defined as the time from the start of treatment to death or the date of the last follow-up, and PFS was defined as the time from the start of treatment to disease progression or death from any cause or the date of the last follow-up.

We first utilized the OS and genomic data of patients from the Liu cohort to construct a nomogram and validated it using the Shoushtari cohort. We also explored the predictive performance of the first nomogram compared with the biomarker TMB. We counted the total number of nonsynonymous mutations of metastatic melanoma patients and the TMB was calculated by,

$$\:TMB=\frac{Total\:nonsynonymous\:mutations}{Exome\:size\:(default:38Mb)}$$

The TMB was reported as mutations per megabase (mutations/megabase, mut/Mb) using the R package ‘maftools’ [22] (version 2.8.05). Additionally, we employed the TCGA cohort and the MSK cohort, consisting of metastatic melanoma patients who did not receive PD-1 inhibitors, as negative controls to assess whether this nomogram could serve as a prognostic model.

In order to further investigate the influences of the predictive model, we proceeded to construct two additional nomograms. The second nomogram was developed using the Liu cohort as the training cohort, but using PFS as the survival outcomes. Conversely, the third nomogram was built using the Shoushtari cohort as the training cohort and using OS as the survival outcomes.

### Statistical analysis

Statistical analysis was performed using R (version 4.1.0; http://www.Rproject.org). The comparisons of clinical characteristics and mutant genes between the training and validation cohorts were done with the $\:{\chi\:}^{2}$ test or Fisher test (using the *chisq.test* or *fisher.test* function in *stats* package, version 4.1.0). We used the *maftools* package (version 2.8.05) calculated TMB for each metastatic melanoma patients. We built a multivariate Cox regression model using genomic mutant, CNV amplified and deleted genes selected from univariate Cox regression analysis (using *survival* package, version 3.2–13). Backward selection was used to test their independent significance. We then used the multivariate Cox regression model to build a nomogram showing the one-year and three-year survival rates of the patients in training cohort (using the *rms* package, version 6.2-0). A risk score for each patient in all four cohorts was calculated using the nomogram, and the calibration curves (1- and 3-year), the decision curve analysis and the concordance index (C-index) was used to demonstrate its performance (using the *rms* package, version 6.2-0; *rmda* package, version 1.6; the *riskRegression* package, version 2023.03.22). Finally, we selected the optimal cut-point for risk score and TMB using maximally selected rank statistics (using *survminer* package, version 0.4.9). The OS and PFS were calculated using the Kaplan-Meier method and log-rank test (using *survival* package, version 3.2–13; *ggplot2* package, version 3.3.5). We also used the spearman rank correlation to investigate the relationship between risk score and TMB. All statistical tests were two-sided, and *P* < 0.05 was statistically significant.

## Result

### Clinical characteristics

The simple clinical characteristics of the Liu and Shoushtari cohorts were shown in Table 1. There were 97 (73.93%) and 122 (79.74%) cutaneous melanomas in the Liu and Shoushtari cohorts, which was almost same (*P* = 0.1753) between both cohorts. Meanwhile, there were no significant differences in ECOG score or metastatic sites between the two patient cohorts.

**Table 1:** Clinical characteristics between the Liu and Shoushtari cohorts

Melanoma Subtype, *n*(%)	Liu cohort (*N* = 133)	Shoushtari cohort (*N* = 153)	χ2	*P*
			1.84	0.1753
Cutaneous	97(72.93)	122(79.74)		
Non- Cutaneous	36(27.07)	31(20.26)		
ECOG, n(%)			–	0.0742
0	94(72.31)	91(59.48)		
1	33(25.38)	52(33.99)		
2	2(1.54)	8(5.23)		
3	1(0.77)	2(1.31)		
LDH Normal, n(%)	69(51.88)	61(39.87)	4.14	0.0419
Lung Metastatic, n(%)	50(37.59)	74(48.37)	3.36	0.0667
Bone Metastatic, n(%)	102(76.69)	123(80.39)	0.58	0.4461
Liver Metastatic, n(%)	76(57.14)	129(84.31)	25.87	< 0.0001

–: Fisher exact test

### Development of the first nomogram

We first evaluated the genomic heterogeneity of patients from the Liu and Shoushtari cohorts using UMAP (*umap* function in *umap* package, version 0.2.10.0), t-SNE (*Rtsne* function in *Rtsne* package, version 0.17) and PCA (*prcomp* function in *stats* package, version 4.1.0). As shown in Supplemental Fig. 2, there was no significant heterogeneity between the two cohorts.

The univariate Cox regression analysis was performed to screen mutated genes, amplified genes and deleted genes associated with OS in patients with metastatic melanoma undergoing anti-PD-1 monotherapy. From a total of 588 genes, 9 candidate genes selected based on a significance threshold of *P* < 0.05 in the univariate Cox regression model (Supplemental Table 1). Subsequently, we conducted a multivariate Cox regression analysis with backward selection using these candidate genes. The results of multivariate analysis were presented in Supplemental Table 2. Our analysis revealed that five genes, namely NID1 amplification, TYRP1 deletion, mutations of PIK3C2G, FLT1 and IKZF1, were independently associated with OS in metastatic melanoma patients undergoing anti-PD-1 monotherapy. The frequency of variation in these genes between two cohorts was shown in the Supplemental Table 3.

We then constructed the first nomogram (Supplemental Fig. 3) to identify the one-year, three-year OS of metastatic melanoma patients under anit-PD-1 monotherapy based on the multivariate Cox regression model above. The number 0 designations above the lines for each gene means wild type and 1 means mutant or CNV. Additionally, we provided the corresponding points (risk score) for the wild type or mutant/CNV status of each gene in Supplemental Table 4. The relevant results of this nomogram and risk identification for melanoma patients also could be accessed directly at https://yyw0505.shinyapps.io/nomogram_app/.

We first evaluated the predictive performance of the nomogram for 1-year and 3-year OS and PFS in patients from the Liu cohort using calibration curves, which were shown in Supplemental Fig. 4, demonstrating that our nomogram exhibited excellent predictive performance. The decision curve analysis shown this nomogram could significantly improve the 3-year OS and PFS benefits for melanoma patients (Supplemental Fig. 4c and 4f). And the C-index indicated that this nomogram achieved superior predictive consistency for OS compared to PFS (Supplemental Table 5).

**Fig. 1:**
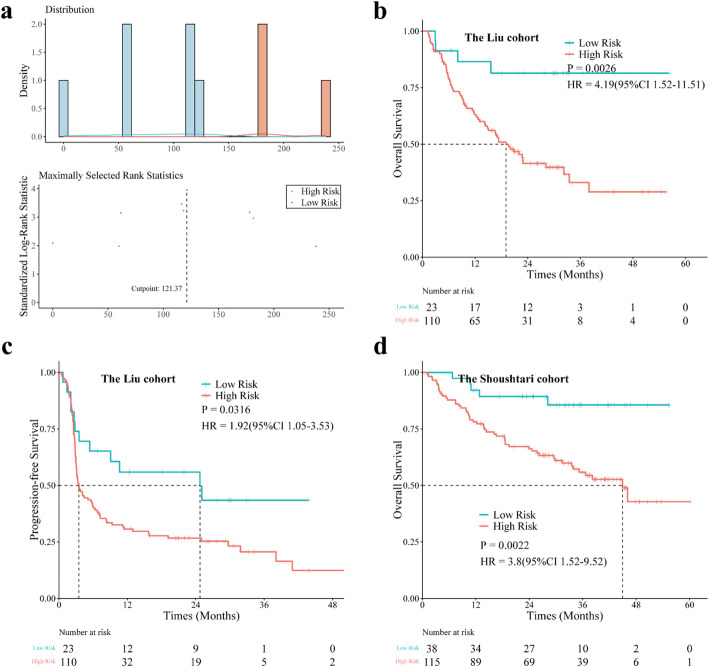
The risk score in the Liu cohort and the Shoushtari cohort of metastatic melanoma patients with anti-PD-1 treatment. **a** The optimal cut-point of the risk score based on overall survival in the Liu cohort to group patients into High-Risk and Low-Risk groups. Kaplan-Meier estimate of **b** overall survival and **c** progression-free survival between High-Risk and Low-Risk groups in the Liu cohort. **d** Kaplan-Meier estimates of overall survival between High-Risk and Low-Risk groups in the Shoushtari cohort. *HR* hazard ratio

We further calculated a risk score for each patient in both cohorts using the first nomogram. The risk scores ranged from 0 to 278.136 in Liu cohort and from 0 to 338.275 in the Shoushtari cohort. Notably, we observed a significant negative correlation between the risk score and TMB in both the Liu cohort (*r*_*s*_ = – 0.5140, *P* < 0.0001, Supplemental Fig. 5a) and the Shoushtari cohort (*r*_*s*_ = – 0.4980, *P* < 0.0001, Supplemental Fig. 5b). The optimal cutoff value for the risk score, determined by maximally selected rank statistics, was found to be 121.37 (Fig. 1a). Accordingly, patients with risk score ≥ 121.37 were classified into High-Risk group, while those below the cutoff were assigned to the Low-Risk group. In the Liu cohort, a total of 23 (17.29%) patients were assigned to the Low-Risk group, while 110 (82.71%) patients were grouped into the High-Risk group. The Kaplan-Meier survival curves demonstrated that patients in the High-Risk group had a significantly shorter OS (19.1 months vs. not reached, HR 4.19, 1.52–11.51, *P* = 0.0026, Fig. 1b) and PFS (3.5 vs. 24.7 months, HR 1.92, 1.05–3.53, *P* = 0.0316, Fig. 1c) than those in Low-Risk group.

### Validation of the first nomogram

To validate the predictive performance of the first nomogram, we initially performed the calibration curves, decision curve analysis and C-index utilized the Shoushtari cohort, the results also demonstrated good predictive and decisive performance on the validation cohort (Supplemental Fig. 6 and Supplemental Table 5). Based on the risk score and its cutoff value, there were 38 (24.84%) patients grouped into the Low-Risk group and 115 (75.26%) patients grouped into the High-Risk group. And the metastatic melanoma patients with high risk also had shorter OS (44.9 months vs. not reached, HR 3.80, 1.52–9.52, *P* = 0.0022, Fig. 1d) compared to those in the Low-Risk group.

**Fig. 2:**
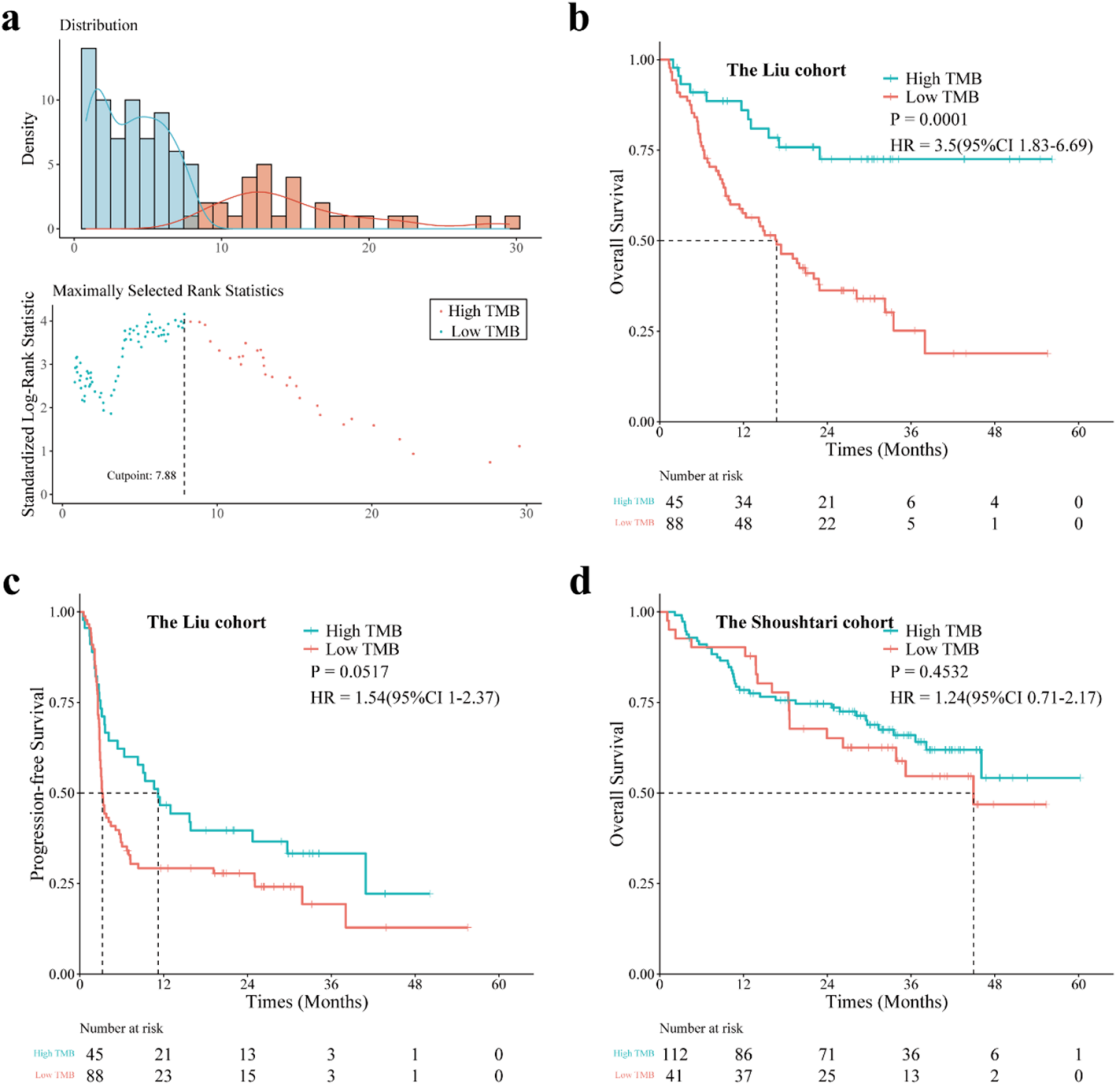
The TMB in the Liu cohort and the Shoushtari cohort of metastatic melanoma patients with anti-PD-1 treatment. **a** The optimal cut-point of the TMB based on overall survival in the Liu cohort to group patients into High-Risk and Low-Risk groups. Kaplan-Meier estimate of **b** overall survival and **c** progression-free survival between High-Risk and Low-Risk groups in the Liu cohort. **d** Kaplan-Meier estimates of overall survival between High-Risk and Low-Risk groups in the Shoushtari cohort. *TMB* tumor mutation burden. *HR* hazard ratio

In order to compare the predictive performance of the first nomogram with TMB, we also employed the maximally selected rank statistics to determine the optimal cutoff for TMB, which was found to be 7.88 (Fig. 2a). Subsequently, patients in both the Liu cohort and Shoushtari cohort were grouped into High-TMB group (TMB ≥ 7.88) and Low-TMB group (TMB < 7.88). The Kaplan-Meier curve demonstrated that patients in the Low-TMB group had a significantly shorter OS (16.7 months vs. not reached, HR 3.50, 1.83–6.69, *P* = 0.0001, Fig. 2b) in the Liu cohort. However, no significant difference was observed in PFS (3.22 vs. 11.20 months, HR 1.54, 1.00-2.37, *P* = 0.0517, Fig. 2c) between the Low-TMB and High-TMB groups in the Liu cohort. Furthermore, in the Shoushtari cohort, there was not significantly different in OS (44.90 months vs. not reached, HR 1.54, 1.00-2.37, *P* = 0.4532, Fig. 2d) between Low-TMB and High-TMB groups.

We further divided metastatic melanoma patients into High-TMB group and Low-TMB group using the criterion TMB ≥ 10, which had been approved by the FDA. The results demonstrated that patients in the Low-TMB group had shorter OS (17.4 months vs. not reached, HR 2.91, 1.51–5.56, *P* = 0.0007, Supplemental Fig. 7a) compared to those in the High-TMB group. However, no significant difference was observed in PFS (3.3 vs. 11.0 months, HR 1.41, 0.90–2.20, *P* = 0.1318, Supplemental Fig. 7b) in the Liu cohort and OS (44.9 months vs. not reached, HR 1.41, 0.83–2.40, *P* = 0.2023, Supplemental Fig. 7c) in the Shoushtari cohort.

### Relationship between the identified high-risk and melanoma subtypes

As shown in Table 1, the study population primarily consisted of cutaneous melanoma patients. Therefore, we further investigated the high-risk result identified by this nomogram was confounded by melanoma subtype (cutaneous vs. non-cutaneous). As shown in Supplemental Fig. 8a, there were 76 (78.35%) and 34 (94.44%) High-Risk patients identified from the cutaneous and non-cutaneous melanoma patients in the Liu cohort. The same results were observed in the Shoushtari cohort, with 88 (72.13%) and 27 (87.1%) High-Risk patients identified from the cutaneous and non-cutaneous subtype (Supplemental Fig. 8b). The results indicated that a higher proportion of patients with cutaneous melanoma were classified as High-Risk.

We further compared the OS across melanoma subtypes and found that non-cutaneous melanoma patients demonstrated shorter OS (8.82 months vs. 33.50 months, HR 2.21, 1.35–3.64, *P* = 0.0013, Supplemental Fig. 8c) in the Liu cohort. However, in the Shoushtari cohort, there was no significant difference in OS (46.1 months vs. not reached, HR 1.03, 0.54–1.95, *P* = 0.9252, Supplemental Fig. 8d) between cutaneous and non-cutaneous melanoma patients. All these results indicating that, although the predictions of this nomogram correlated with melanoma subtypes, the High-Risk versus Low-Risk patients identified by our model demonstrated greater clinical significance than the subtypes (cutaneous vs. non-cutaneous).

### Performance of the first nomogram in cutaneous metastatic melanoma

**Fig. 3:**
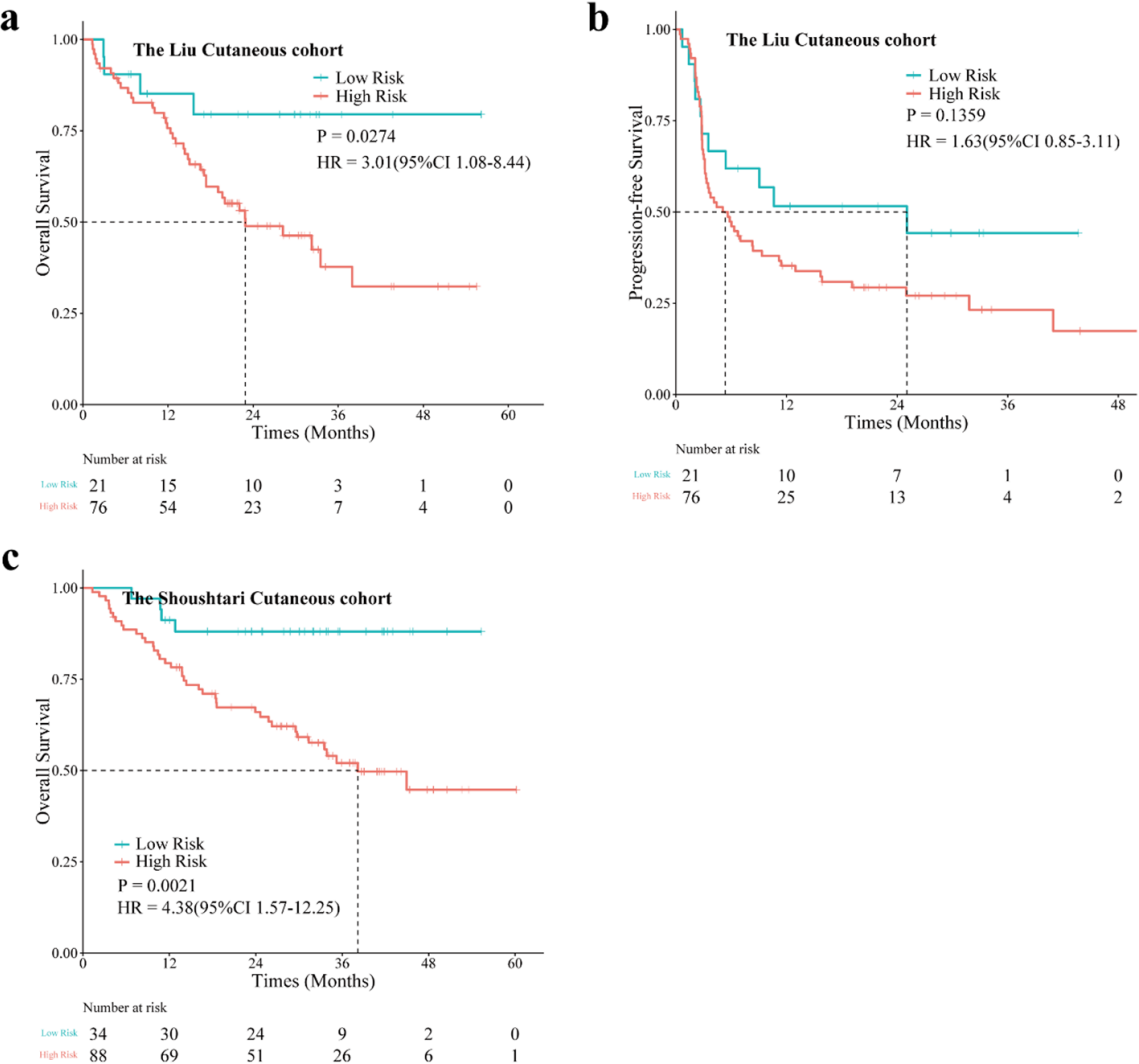
Kaplan-Meier estimates of patients with cutaneous metastatic melanoma after anti-PD-1 treatment. Kaplan-Meier curves of **a** overall survival and **b** progression-free survival for patients in the Liu cohort. Kaplan-Meier curves of **c** overall survival for patients in the Shoushtari cohort

We further explored performance of the first nomogram to predict survival in patients with cutaneous metastatic melanoma who underwent anti-PD-1 monotherapy. The Kaplan-Meier survival curves demonstrated that patients in the High-Risk group had a significantly shorter OS (22.9 months vs. not reached, HR 3.01, 1.08–8.44, *P* = 0.0274, Fig. 3a) and PFS (5.37 vs. 25.07 months, HR 1.63, 0.85–3.11, *P* = 0.1359, Fig. 3b) than those in the Low-Risk group. Similarly, in the Shoushtari cohort, patients with cutaneous metastatic melanoma in the High-Risk group also exhibited a significantly shorter OS (38.2 months vs. not reached, HR 4.38, 1.57–12.25, *P* = 0.0021, Fig. 3c) than patients in the Low-Risk group.

### Predictive and prognostic role of the first nomogram

**Fig. 4:**
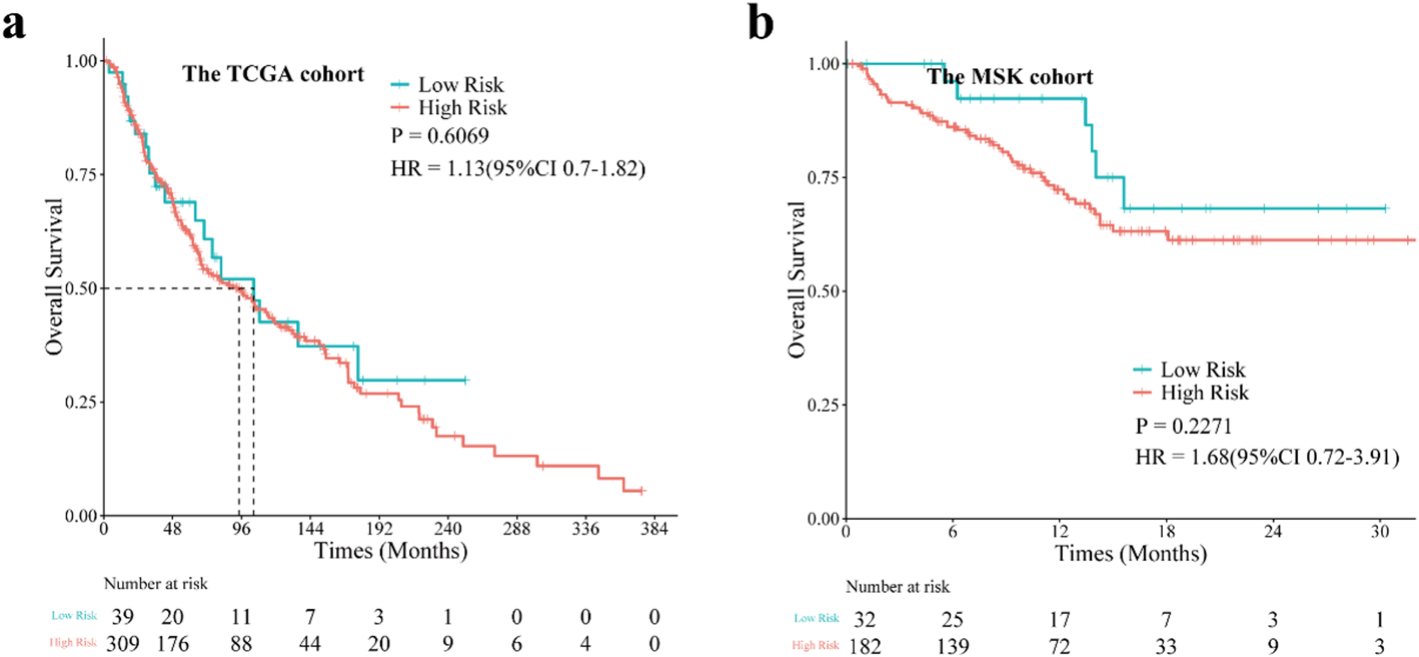
Kaplan-Meier estimates of metastatic melanoma patients without anti-PD-1 treatment. Kaplan-Meier curves of overall survival for metastatic melanoma patients without anti-PD-1 therapy in **a** TCGA cohort and **b** MSK cohort. *TCGA* The Cancer Genome Atlas, *MSK* Memorial Sloan Kettering

To further explore whether the first nomogram could serve as a prognostic model for patients with metastatic melanoma, we utilized the TCGA cohort and the MSK cohort as negative controls, which consisted with metastatic melanoma patients without anti-PD-1 monotherapy. Using the nomogram, we identified 39 (11.21%) low-risk patients and 309 (88.79%) high-risk patients in the TCGA cohort. Surprisingly, there was no significant difference in OS (94.3 vs. 104.5 months, HR 1.13, 0.70–1.82, *P* = 0.6069, Fig. 4a) between the two groups. Similarly, in the MSK cohort, there were 32 (14.95%) patients assigned to the High-Risk group and 182 (85.05%) patients assigned to the Low-Risk group. And we also observed there was no significant difference in OS (HR 1.68, 0.72–3.91, *P* = 0.2271, Fig. 4b) between the High-Risk group and Low-Risk group. Therefore, we considered this nomogram to be a predictive model for survival outcomes in melanoma patients with anti-PD-1 monotherapy, rather than a prognostic model.

### The second nomogram based on PFS of the Liu cohort

In the aforementioned study, we made an interesting observation that the first nomogram, which was developed based on OS, could effectively identify PFS in metastatic melanoma patients undergoing anti-PD-1 monotherapy. Motivated by this finding, we proceeded to construct the second nomogram based on the PFS from the Liu cohort to explore its ability to predict patients’ OS. We first selected 17 candidate genes from 588 genes using univariate Cox regression analysis (Supplemental Table 6), and screened 4 genes (mutations of TYRP1 and IL7R, deletion of TYRP1 and amplification of NID1) used to construct the second nomogram based on multivariate Cox regression analysis with backward selection (Supplemental Table 7). The second nomogram was shown in Supplemental Fig. 9 and the risk scores of each gene were shown in Supplemental Table 8. We further used the second nomogram to predict risk scores for each patient. The risk scores for patients in the Liu cohort ranged from 0 to 210.18, while ranged from 0 to 265.85 in the Shoushtari cohort.

**Fig. 5:**
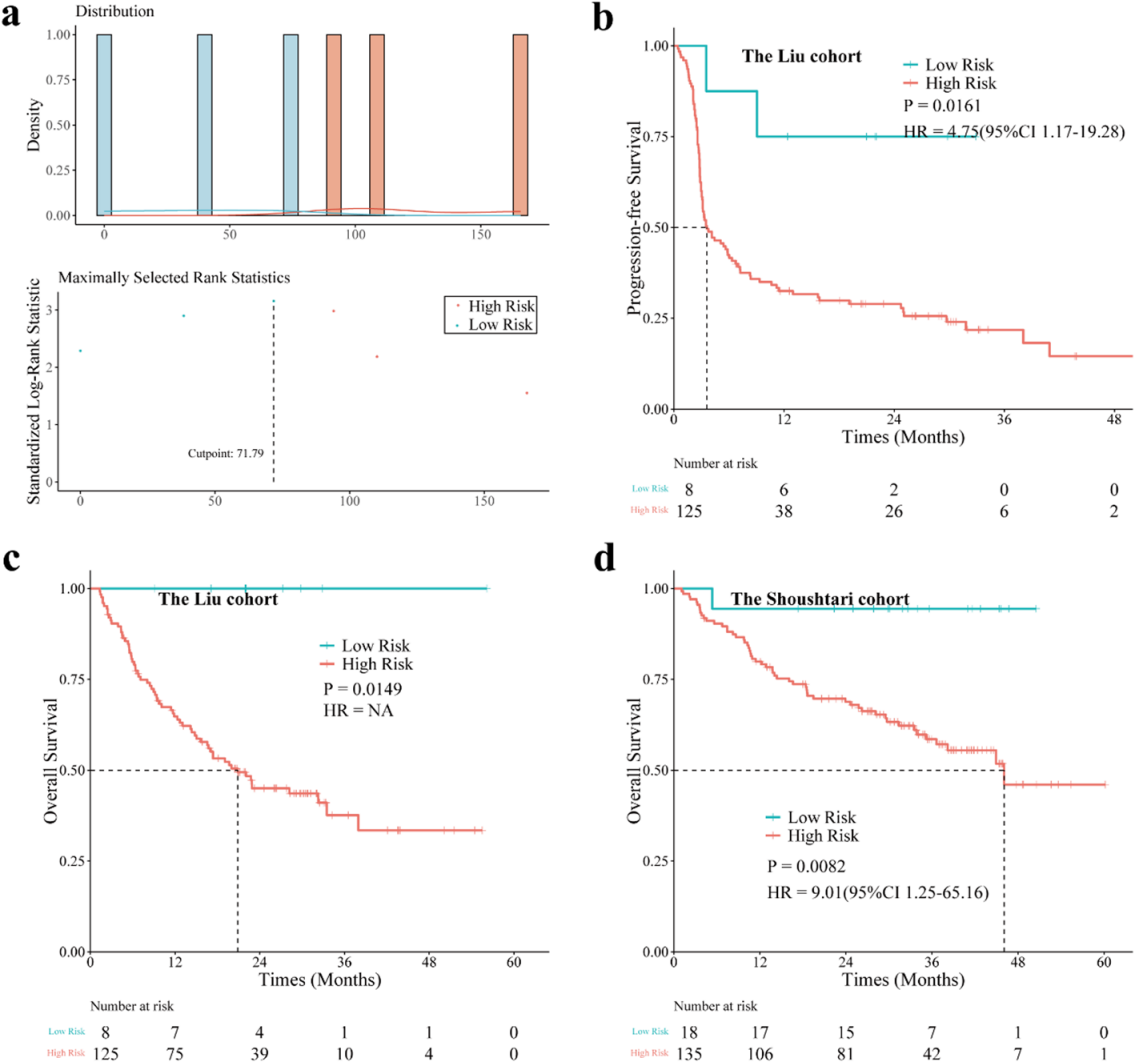
The risk score in the Liu cohort and the Shoushtari cohort of metastatic melanoma patients with anti-PD-1 treatment obtained from the second nomogram. **a** The optimal cut-point of the risk score based on progression-free survival in the Liu cohort to group patients into High-Risk and Low-Risk groups. Kaplan-Meier estimate of **b** progression-free survival and **c** overall survival between High-Risk and Low-Risk groups in the Liu cohort and **d** overall survival between High-Risk and Low-Risk groups in the Shoushtari cohort. *HR* hazard ratio

As shown in Fig. 5a, the optimal cutoff value for discrimination of High-Risk group and Low-Risk group using the maximally selected rank statistics was 71.79. In the Liu cohort, 8 (6.02%) patients were classified into the High-Risk group, while 125 (93.98%) were classified into the Low-Risk group. In the Shoushtari cohort, 18 (11.76%) patients were categorized as High-Risk, and 135 (88.24%) patients were classified as Low-Risk. We observed the patients in High-Risk group exhibited a significantly shorter PFS (3.60 months vs. not reached, HR 4.75, 1.17–19.28, *P* = 0.0161, Fig. 5b) and OS (3.60 months vs. not reached, *P* = 0.0161, Fig. 5c) in the Liu cohort. In the Shoushtari cohort, we also found that patients in the High-Risk group had a shorter OS (46.10 months vs. not reached, HR 9.01, 1.25–65.16, *P* = 0.0161, Fig. 5d) compared to those in the Low-Risk group. The Kaplan-Meier survival curves between the High-Risk and Low-Risk groups of cutaneous metastatic melanoma were demonstrated in Supplemental Fig. 10, and those results showed that patients in High-Risk group still had significantly poor OS and PFS in both cohorts. These results demonstrated that the second nomogram developed based on the Liu cohort’s PFS could also identify patients at risk for OS from the Shoushtari cohort, but its predictive performance was inferior to the first nomogram. Therefore, we recommend utilizing the second nomogram to identify the PFS in melanoma patients undergoing anti-PD-1 monotherapy.

### The third nomogram based on OS of the Shoushtari cohort

Considering there was differences in gene variant frequencies between cohorts, which was shown in Supplemental Table 3 (e.g., TYRP1_del and FLT1_mut), and those variations might affect the accuracy of the results obtained above when using the Shoushtari cohort as a validation set. We further built the third nomogram using OS of the Shoushtari cohort and validated it using the Liu cohort. In the Shoushtari cohort, we filtered out 66 genes according to *P* < 0.05 using univariate Cox regression analysis (Supplemental Table 9). After applying a multivariate Cox regression analysis with a backward selection algorithm, we identified seven genes (mutations of INSR and IL7R, amplification of CR1, HLA-G, HLA-A, HLA-B and HLA-DRB5) to build the third nomogram, and the results of the multivariate Cox regression were shown in Supplemental Table 10. The third nomogram was shown in Supplemental Fig. 11, and the risk score associated with each gene in the third nomogram could be found in Supplemental Table 11. Patients’ risk scores which calculated using the third nomogram were ranged from 174.32 to 309.92 in the Shoushtari cohort, while from 140.06 to 373.20 in the Liu cohort.

**Fig. 6:**
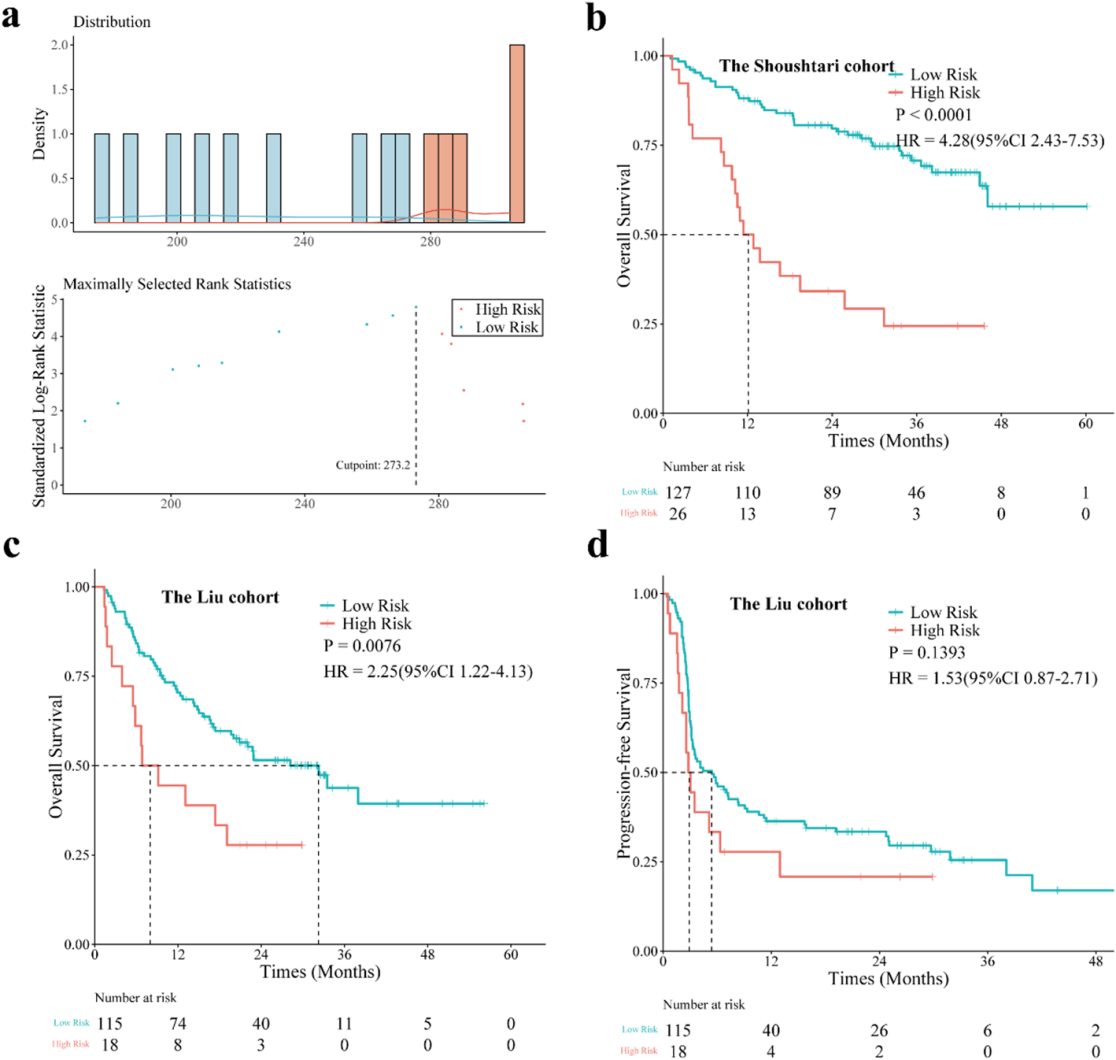
The risk score in the Shoushtari cohort and the Liu cohort of metastatic melanoma patients with anti-PD-1 treatment obtained from the third nomogram. **a** The optimal cut-point of the risk score based on overall survival in the Shoushtari cohort to group patients into High-Risk and Low-Risk groups. Kaplan-Meier estimate of **b** overall survival between High-Risk and Low-Risk groups in the Shoushtari cohort. Kaplan-Meier estimate of **c** overall survival and **d** progression-free survival between High-Risk and Low-Risk groups in the Liu cohort. *HR* hazard ratio

We also used maximally selected rank statistics to select the optimal cutoff value of risk score was 273.2 (Fig. 6a), and there were 127 (83.01%) patients grouped into the High-Rik group (risk score ≥ 273.2) and 26 (16.99%) patients classified into the Low-Risk group (risk score < 273.2) in the Shoushtari cohort. Similarly, 115 patients (86.47%) were grouped into the High-Risk group and 18 (13.53%) in the Low-Risk group. We found the patients in the High-Risk still had shorter OS in the Shoushtari cohort (12.1 months vs. not reached, HR 4.28, 2.43–7.53, *P* < 0.0001, Fig. 6b) and the Liu cohort (8.00 vs. 32.3 months, HR 2.25, 1.22–4.13, *P* = 0.0076, Fig. 6c). However, the PFS (2.91 vs. 5.40 months, HR 1.53, 0.87–2.71, *P* = 0.1392, Fig. 6d) was not significantly different between two groups in the Liu cohort.

We explored the predictive performance of the third nomogram in cutaneous metastatic melanoma patients, those Kaplan-Meier survival curves were demonstrated in Supplemental Fig. 12. The results showed that patients in High-Risk group still had poor OS in both cohorts of cutaneous metastatic melanoma. And the PFS still was not different between two groups in the Liu cohort of cutaneous metastatic melanoma. These results suggested that even with partial differences in gene mutation frequencies between the Liu and Shoushtari cohorts, the results in our study remain accurate and reliable.

## Discussion

Although the PD-1 inhibitors have significantly decreased the mortality of patients with metastatic melanoma, only a relatively small proportion of patients truly benefit from anti-PD-1 monotherapy. Current studies that evaluate the efficacy of anti-PD-1 monotherapy for treating metastatic melanoma primarily focus on surrogate endpoints, such as response assessment using the RECIST criteria. In this study, we developed the first simple nomogram based on NID1 amplification, TYRP1 deletion, mutations of PIK3C2G, FLT1 and IKZF1 to identify the survival outcomes (endpoints) of metastatic melanoma patients with anti-PD-1 monotherapy. The patients in the High-Risk group, as determined by the first nomogram, exhibited shorter OS and PFS under anti-PD-1 monotherapy. Furthermore, this nomogram accurately identified OS and PFS in cutaneous metastatic melanoma patients with anti-PD-1 monotherapy. In contrast to Zhang et al. [16] who utilized whole-exome sequencing data and Zila et al. [17] who employed proteomics data to construct predictive models for identifying melanoma patients’ response to anti-PD-1 monotherapy, the first nomogram in this study only required the detection of mutations or CNVs in five genes to accurately identify OS and PFS in metastatic melanoma patients undergoing anti-PD-1 monotherapy. This simplicity and efficiency make it more likely to be applied in clinical practice.

All the five genes used to construct the first nomogram were associated with metastatic melanoma and its prognosis. For instance, NID1 was identified as a susceptibility locus for cutaneous melanoma and was found to promote lung metastasis of melanoma [23, 24]. Deng et al. [25] reported a significant correlation between TYRP1 and poor prognoses in melanoma, while Rinner et al. [26] revealed that the mutations in PIK3C2G could reinforce DNA repair problems in melanoma. Pala et al. [27] reported an association between FLT1 and melanoma brain metastases, and Yang et al. [28] observed that decreased expression of IKZF1 was significantly contributed to increased melanoma metastasis and associated with poorer OS. Therefore, we utilized metastatic melanoma patients who did not treated with PD-1 inhibitors in the TCGA and MSK cohorts as negative controls to explore whether the first nomogram we developed was a prognostic model for metastatic melanoma patients. The results revealed that there was no significant difference in OS and PFS between patients in the High-Risk and Low-Risk groups identified by the first nomogram in the two cohorts. This finding suggests that this nomogram was a predictive model for survival outcomes in patients with metastatic melanoma undergoing anti-PD-1 monotherapy, rather than a prognostic model for all patients with metastatic melanoma.

Rizvi and Cristescu et al. previously reported that TMB could serve as a biomarker for predicting response to anti-PD-1 monotherapy in melanoma patients, although a standardized cutoff value was lacking. In this study, we identified an optimal cutoff value of 7.88 for TMB using OS from the Liu cohort and categorized patients into the High-TMB and Low-TMB groups. The results demonstrated that patients in the High-TMB group exhibited better OS compared to those in the Low-TMB group in the Liu cohort. However, there was no significant difference in OS between the two groups in the Shoushtari cohort. Additionally, we grouped patients using the TMB ≥ 10 which was provided by the FDA. Similarly, metastatic melanoma patients with TMB ≥ 10 exhibited longer OS in the Liu cohort, but there was no significant difference between patients with TMB ≥ 10 and TMB < 10 groups in the Shoushtari cohort. These results suggested that TMB had limited ability to identify survival of metastatic melanoma patients receiving PD-1 monotherapy. One important reason for this could be that TMB solely emphasized the number of genomic mutations, rather than focusing on specific function of mutations themselves.

Furthermore, we constructed the second nomogram based on PFS of the Liu cohort to assess its ability to predict OS in patients with metastatic melanoma. The results demonstrated that patients in the Low-Risk group, as identified by the second nomogram, exhibited longer PFS and OS compared to those in the High-Risk group in both the Liu cohort and the Shoushtari cohort. Additionally, we developed the third nomogram using OS of the Shoushtari cohort and validated it using the Liu cohort to examine the impact of different training cohorts on prediction performance. Patients in the High-Risk group, as determined by the third nomogram, had shorter OS and PFS than patients in the Low-Risk group. These consistent results were also observed in patients with cutaneous metastatic melanoma. Overall, these findings indicate that both nomograms possessed robust predictive performance in identifying survival outcomes of patients with metastatic melanoma under anti-PD-1 monotherapy.

There were several limitations in this study. Firstly, the sample size of all the four cohorts used in our study were relatively small, which may limit the generalizability of these findings, and only one cohort was utilized for validation of each of the three nomograms. Secondly, in order to establish parsimonious predictive model, we directly employed backward selection to screen genes without focusing on gene interactions and multiple testing, and it might cause some well-known genes (such as BRAF) to be excluded from our model. Thirdly, due to the heterogeneity in the clinical characteristics of melanoma patients in the Liu and Shoushtari cohorts, and considering the complexity of predictive models, all nomograms in this study were constructed using genomic data only. In the future, it would be beneficial to include larger and more diverse cohorts and the biological information (such as pathway, gene interaction) to further assess the prediction performance of the nomograms in identifying survival outcomes in metastatic melanoma patients undergoing anti-PD-1 monotherapy.

In conclusion, all three nomograms developed in this study demonstrated effectiveness in identifying the survival outcomes of metastatic melanoma patients undergoing anti-PD-1 monotherapy. And we provided a web-based tool (https://yyw0505.shinyapps.io/nomogram_app/) to help clinicians directly and quickly determine melanoma patients’ risk scores and risk outcomes under anti-PD-1 monotherapy. The parsimonious and robust predictive performance of these models have the potential to provide significant assistance in the clinical treatment of patients with metastatic melanoma.

## Supplementary Information

Below is the link to the electronic supplementary material.

Supplementary Material 1.

Supplementary Material 2.

## Data Availability

The datasets of patients treated with anti-PD-1 monotherapy in the Liu cohort used in our study are downloaded from the supplementary information at [https://www.nature.com/articles/s41591-019-0654-5#Sect. 32] . The information of patients in the Shoushtari cohort were publicly available in the cBioPortal and could be downloaded at [https://www.cbioportal.org/study/summary? id=mel_mskimpact_2020] . The information of patients in the MSK cohort were also downloaded from the cBioPortal at https://www.cbioportal.org/study/summary? id=msk_impact_2017. The TCGA cohort was downloaded from the UCSC XENA database at https://xenabrowser.net/datapages/?cohort=GDC%20TCGA%20Melanoma%20(SKCM)&removeHub=https%3 A%2 F%2Fxena.treehouse.gi.ucsc.edu%3A443. The data used and/or analyzed during the current study are available from the corresponding author on reasonable request.
