# Supplementary material for: Predicting of survival in metastatic melanoma patients under anti-PD-1 monotherapy using genomic mutation and copy number variation
Source: Discov Oncol. 2026 May 18;17:1007. doi: 10.1007/s12672-026-05221-8 (PMC13350610; doi:10.1007/s12672-026-05221-8)
Supplement: Supplementary file 1 — Supplementary Material 1. [file 12672_2026_5221_MOESM1_ESM.docx]

**Supplemental Material**


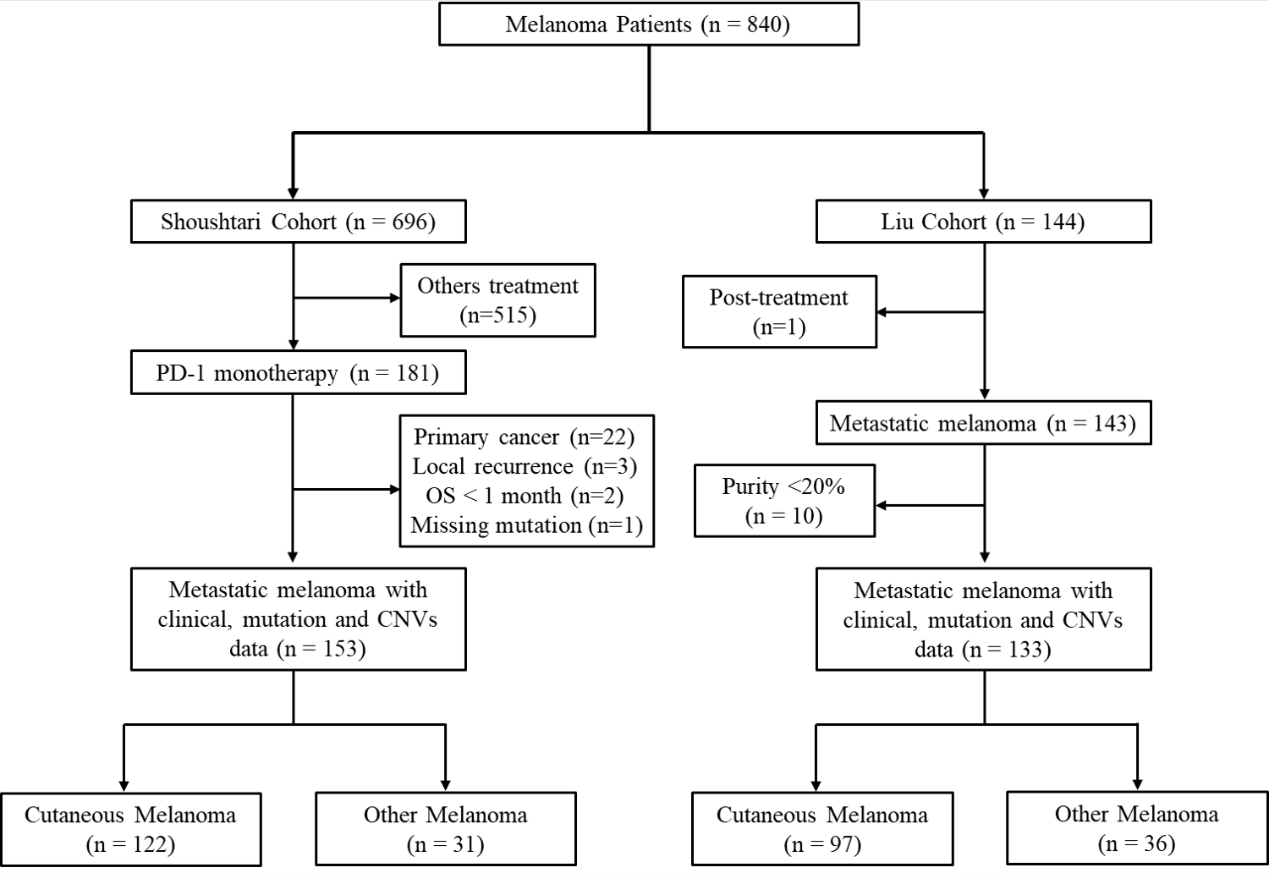


**Supplemental Figure S1.** The process of screening samples. OS, overall survival; CNV, copy number variation.

**
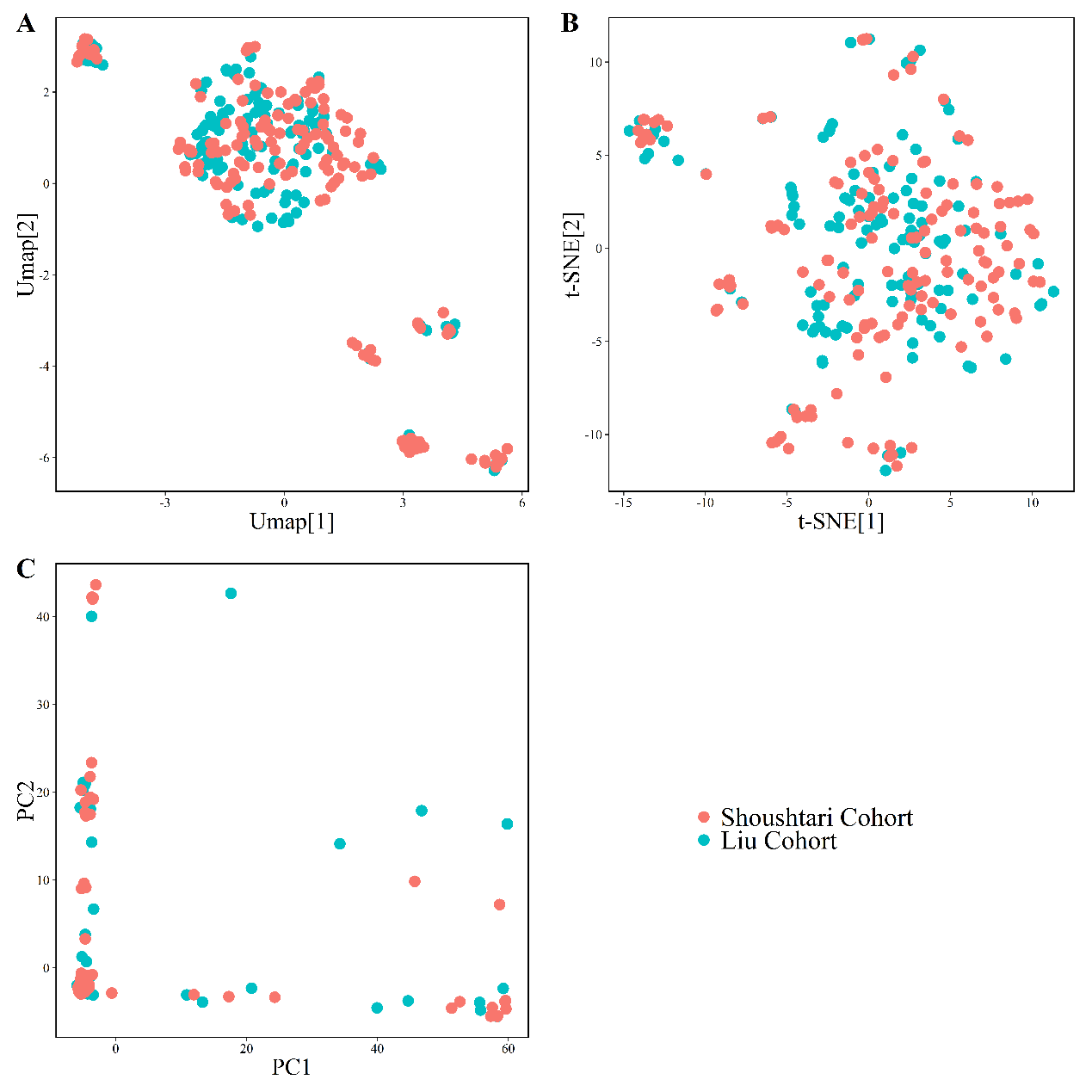
**

**Supplemental Figure S2.** Distribution of patients across the Liu and Shoushtari cohorts. Scatter plot based on (a) the Umap, (b) the t-SNE and (c) the PCA algorithm. Umap, Uniform manifold approximation and projection; t-SNE, t-distributed stochastic neighbor embedding; PCA, principal component analysis.


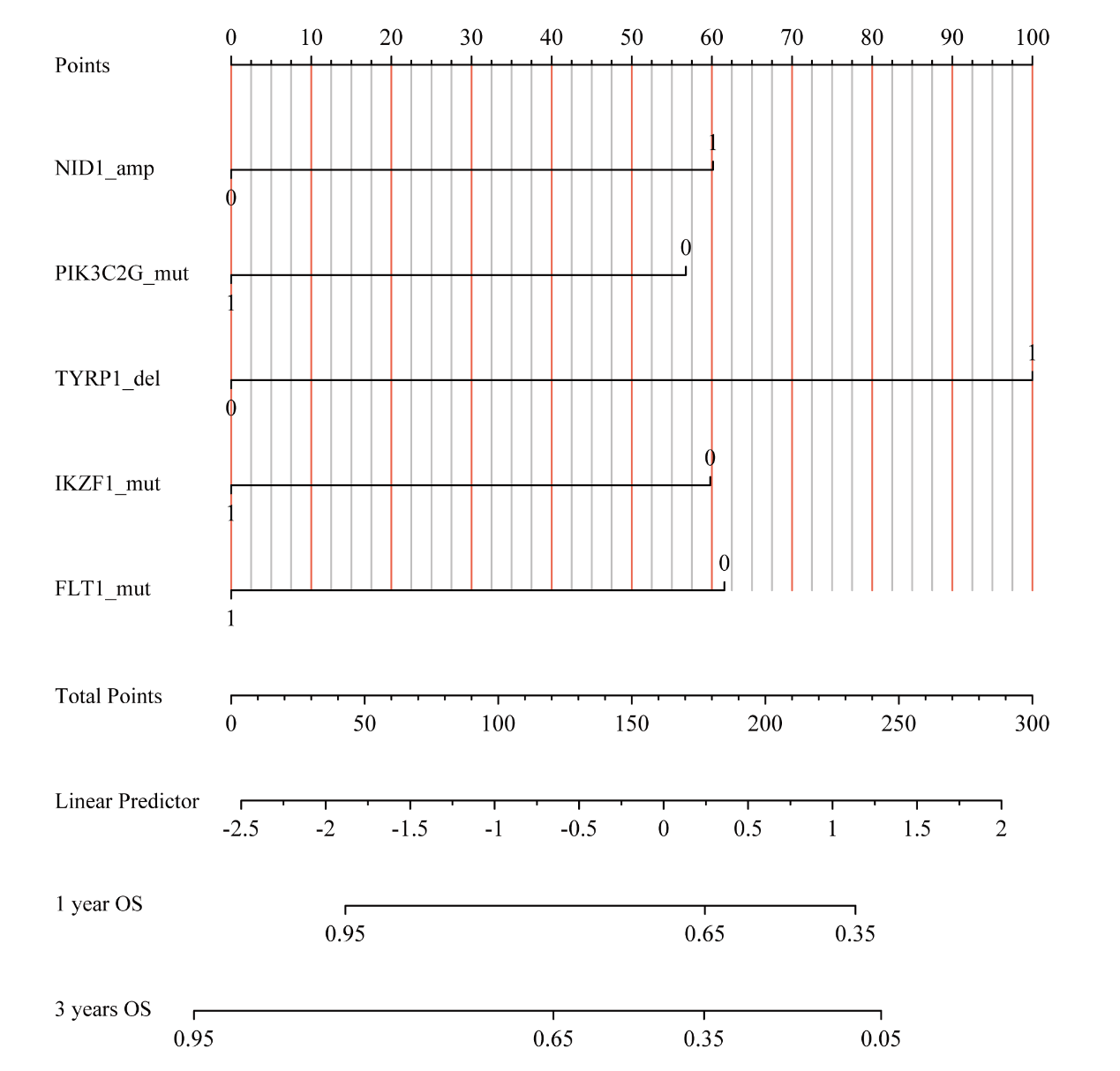


**Supplemental Figure S3.** The first nomogram for predicting the OS in the Liu cohort. OS, overall survival. The number "0" and "1" designations above the lines for each gene mean wild type and mutantion/CNV, respectively. "_mut", "_amp" and "_del" mean genomic mutation, CNV amplification and deletion, such as "NID1_amp" is the amplification of NID1, "PIK3C2G_mut" means the mutation of PIK3C2G gene and "TYRP1_del" means the deletion of TYRP1 gene.

**
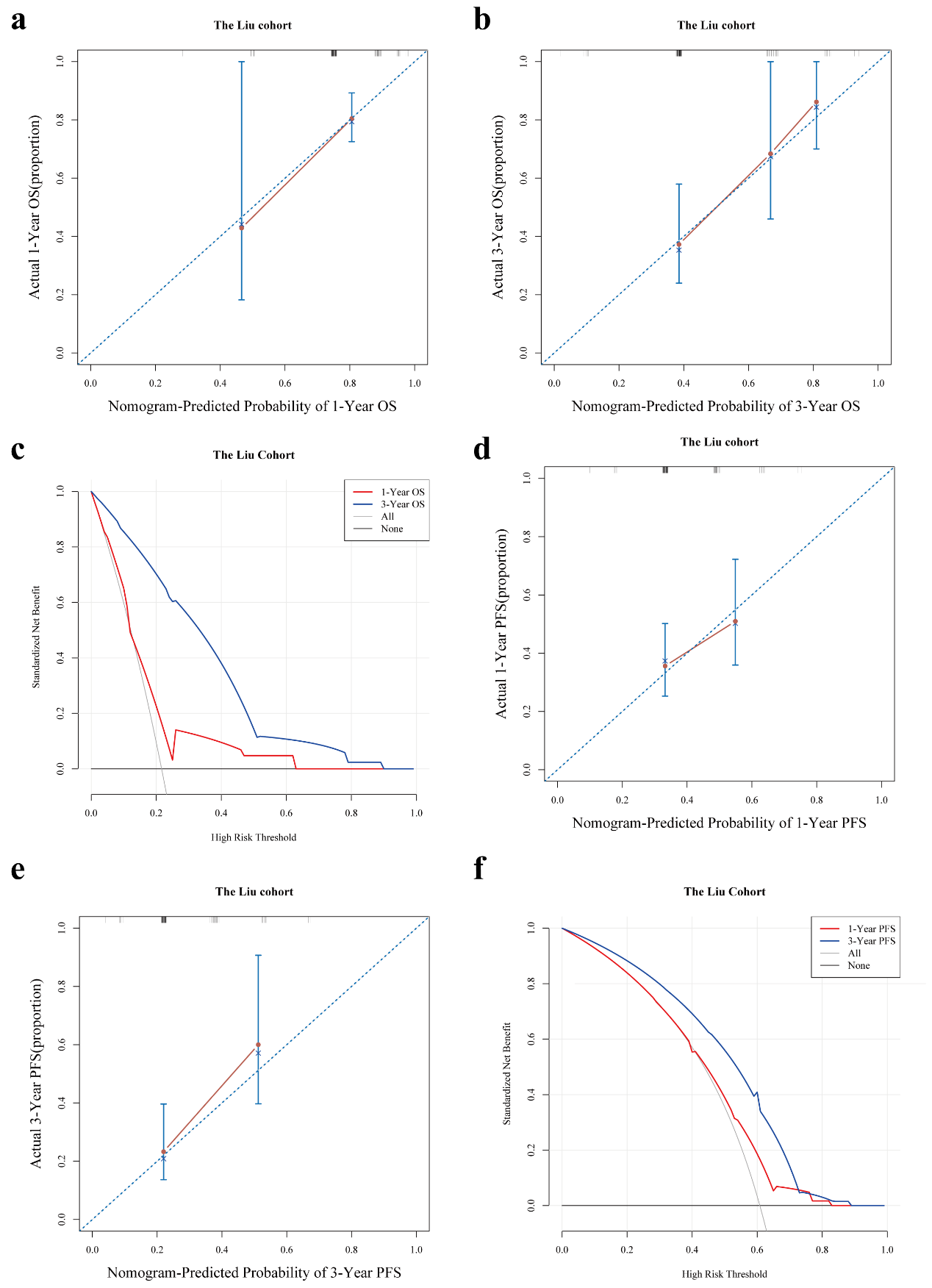
**

**Supplemental Figure S4.** The predictive performance of the first nomogram based on the Liu cohort. The calibration curves of (a) the 1-year OS and (b) the 3-year OS. The decision curve analysis (c) of the 1-year and 3-year OS. The calibration curves of (d) the 1-year OS and (e) the 3-year PFS and (f) the decision curve analysis of PFS.


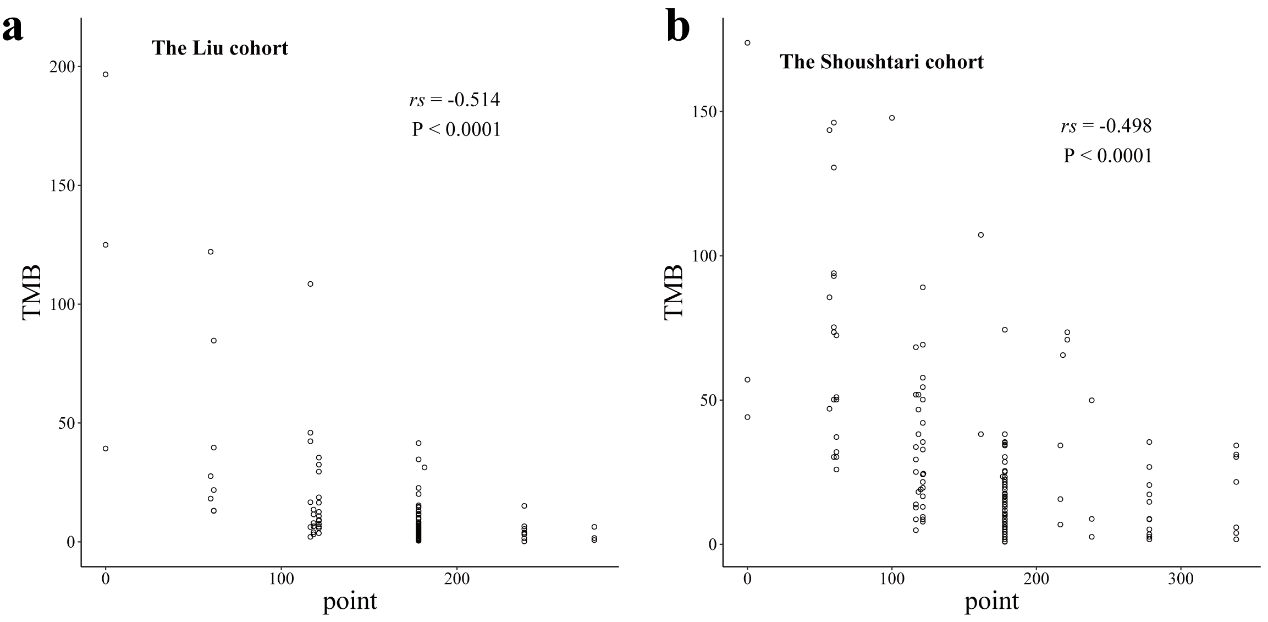


**Supplemental Figure S5.** The relationship between risk score calculated by the first nomogram and TMB. The correlation analysis between risk score and TMB in (a) the Liu cohort and (b) the Shoushtari cohort. The spearman correlation coefficient and *P* value were shown. TMB, tumor mutational burden.


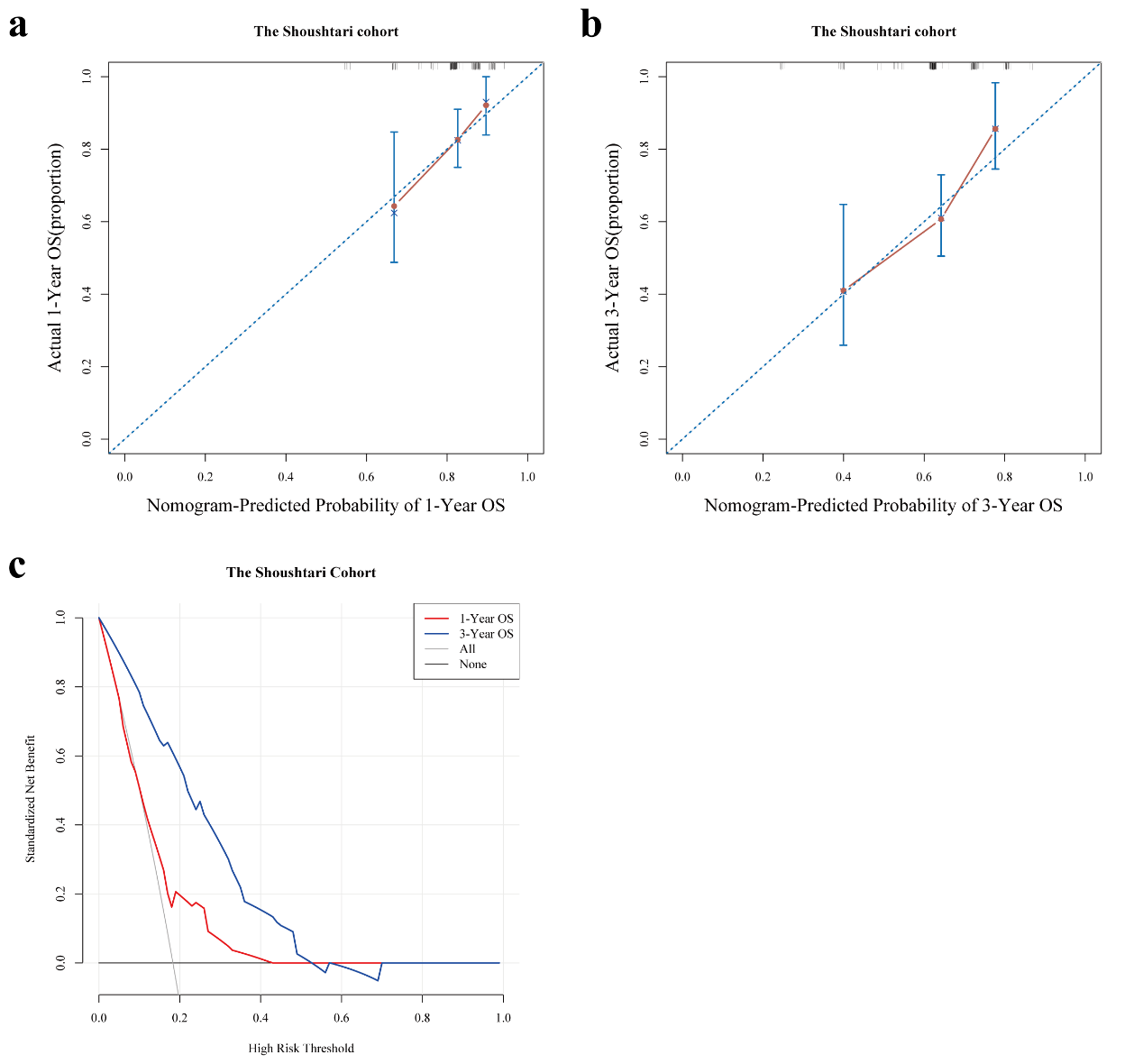


**Supplemental Figure S6.** The predictive performance of the first nomogram based on the Shoushtari cohort. The calibration curves of (a) the 1-year OS and (b) the 3-year OS and the decision curve analysis (c) of the 1-year and 3-year OS.


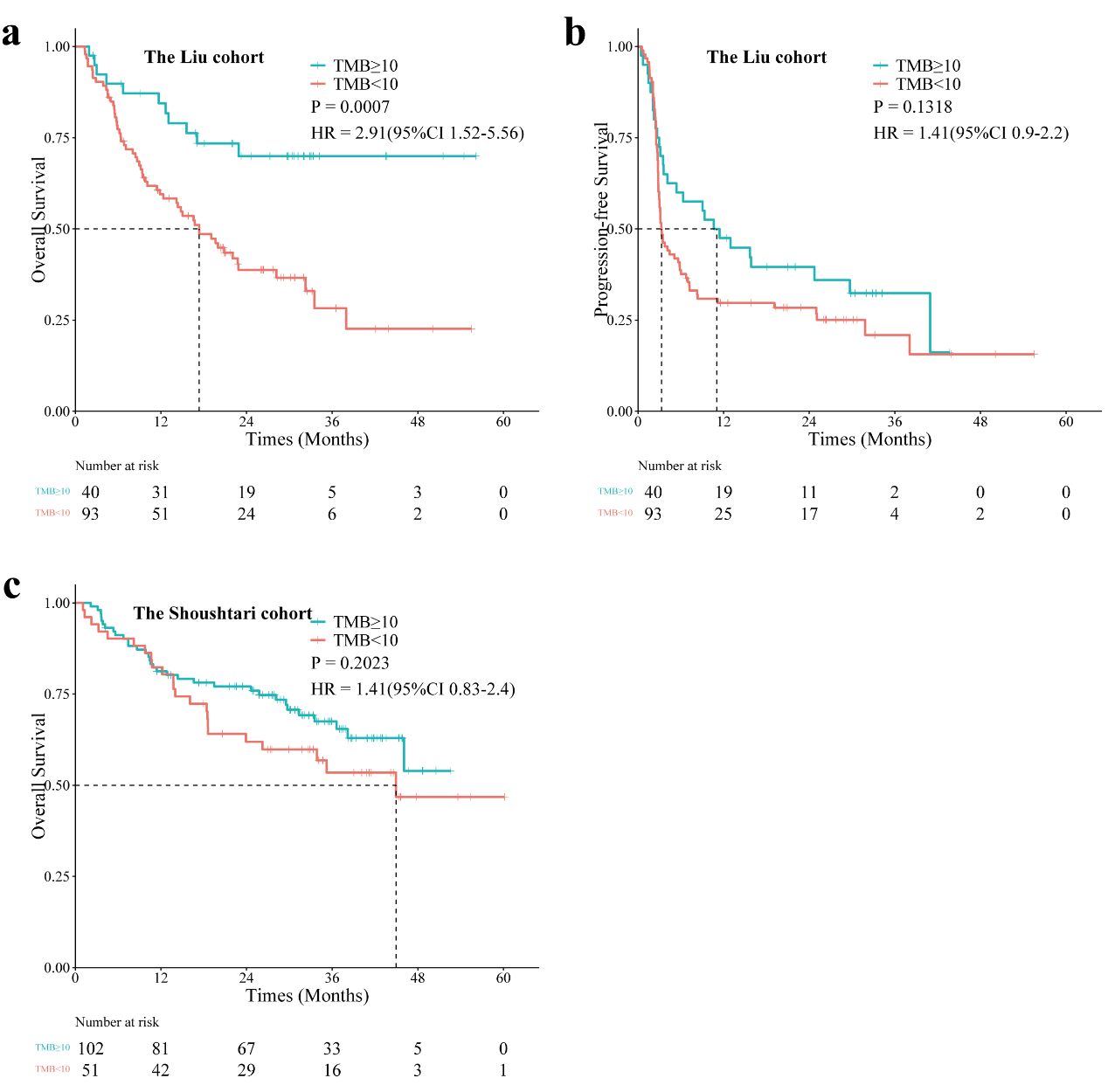


**Supplemental Figure S7.** The Kaplan-Meier curves of metastatic melanoma patients with anti-PD-1 monotherapy when used TMB ≥ 10 mutations/megabase as grouping criteria. Kaplan-Meier estimate of (a) overall survival and (b) progression-free survival between High-TMB and Low-TMB groups in the Liu cohort. Kaplan-Meier estimates of (c) overall survival between High-TMB and Low-TMB groups in the Shoushtari cohort. TMB, tumor mutational burden; HR, hazard ratio.


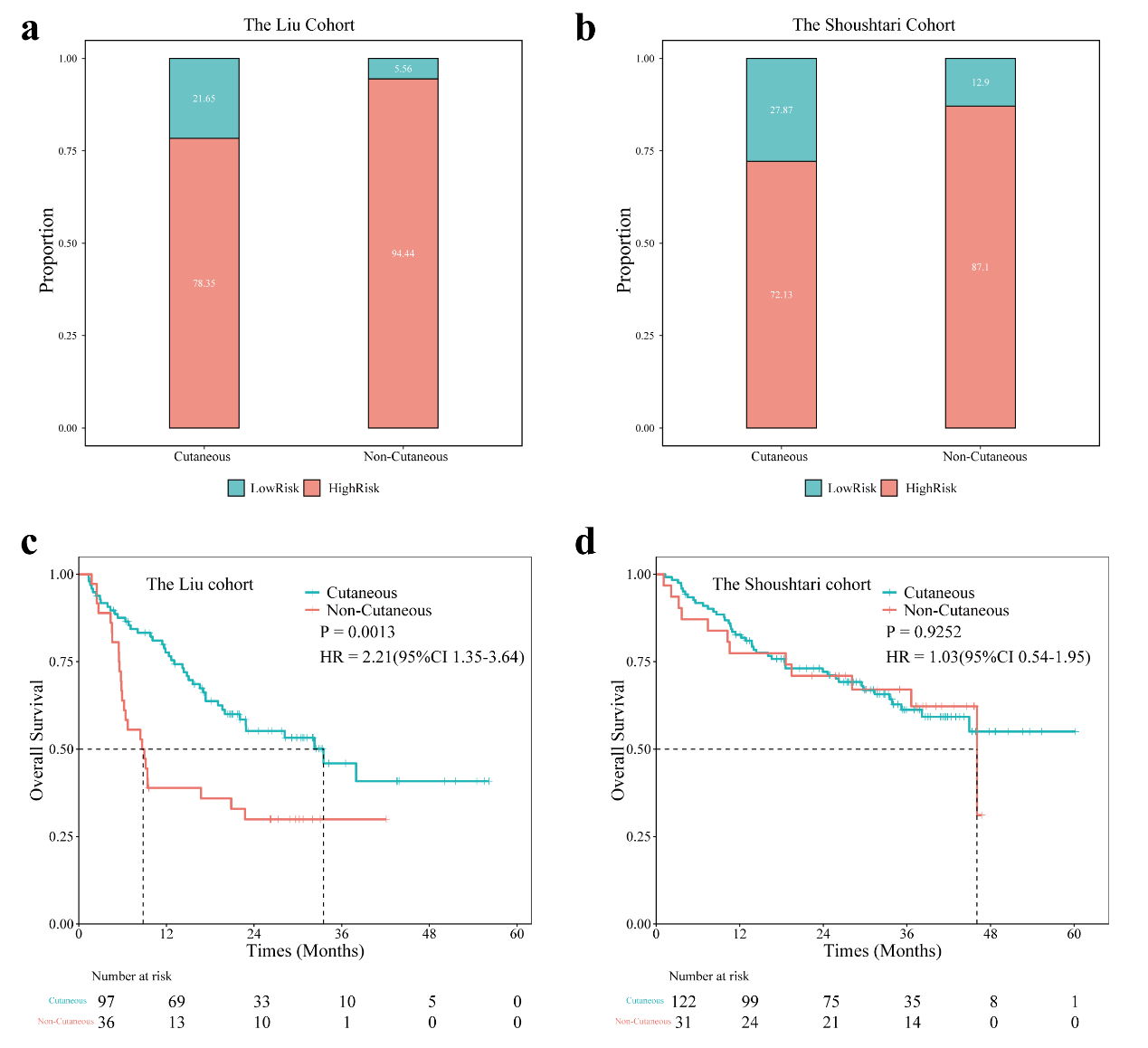


**Supplemental Figure S8.** The relationship between the melanoma subtypes and the high-risk identified by the first nomogram. The percentage of High-Risk and Low-Risk patients identified by the first nomogram across different melanoma subtypes in (a) the Liu cohort and (b) the Shoushtari cohort. Kaplan-Meier estimates of overall survival between cutaneous and non-cutaneous melanoma patients in (c) the Liu cohort and (d) the Shoushtari cohort. HR, hazard ratio.


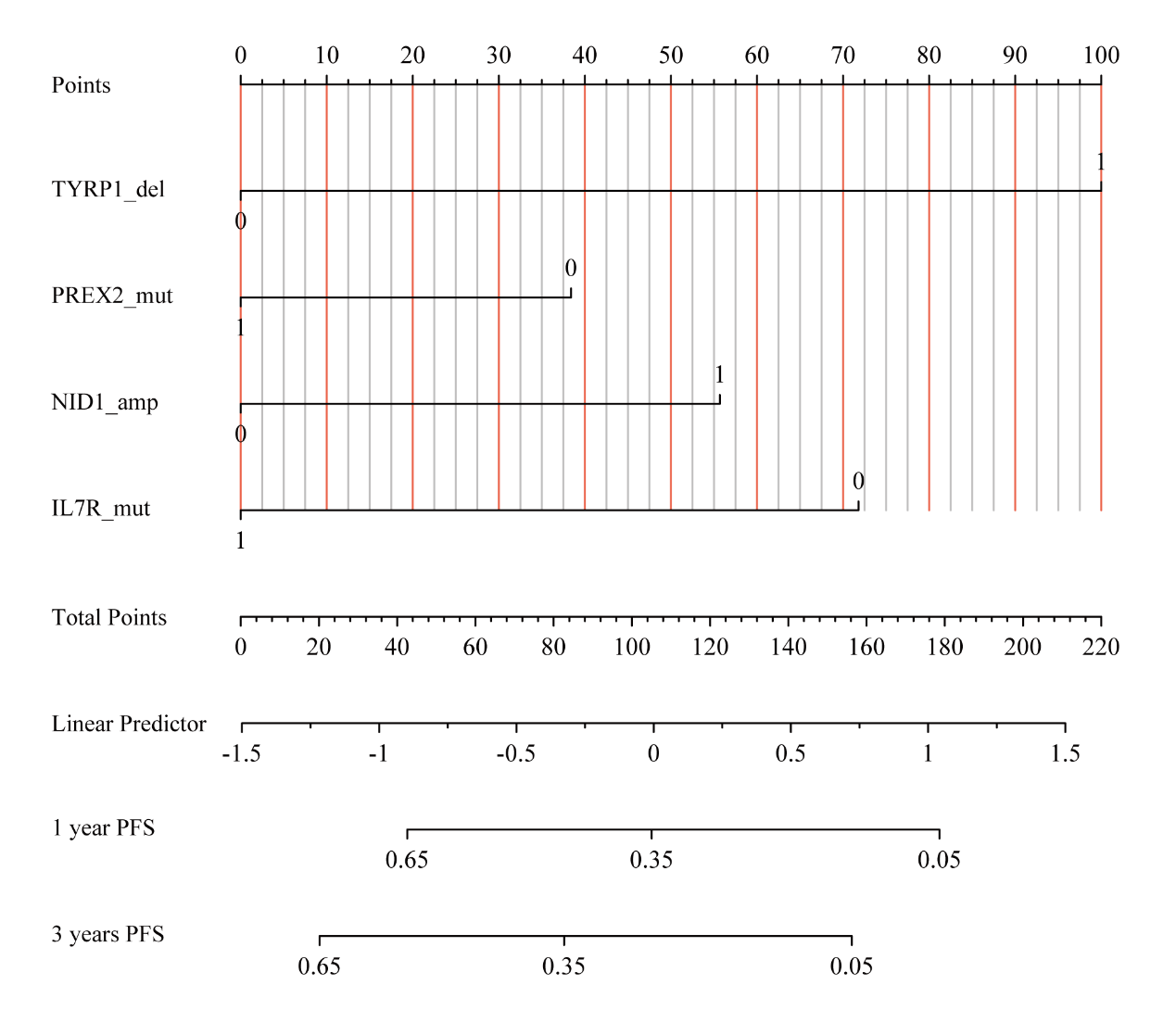


**Supplemental Figure S9.** The second nomogram using PFS of the Liu cohort. The number "0" and "1" designations above the lines for each gene mean wild type and mutant, respectively. "_mut", "_amp" and "_del" mean genomic mutation, CNV amplification and deletion, such as "TYRP1_del" means the deletion of TYRP1 gene, "PREX2_mut" means the mutation of PREX2 gene and "NID1_amp" is the amplification of NID1.


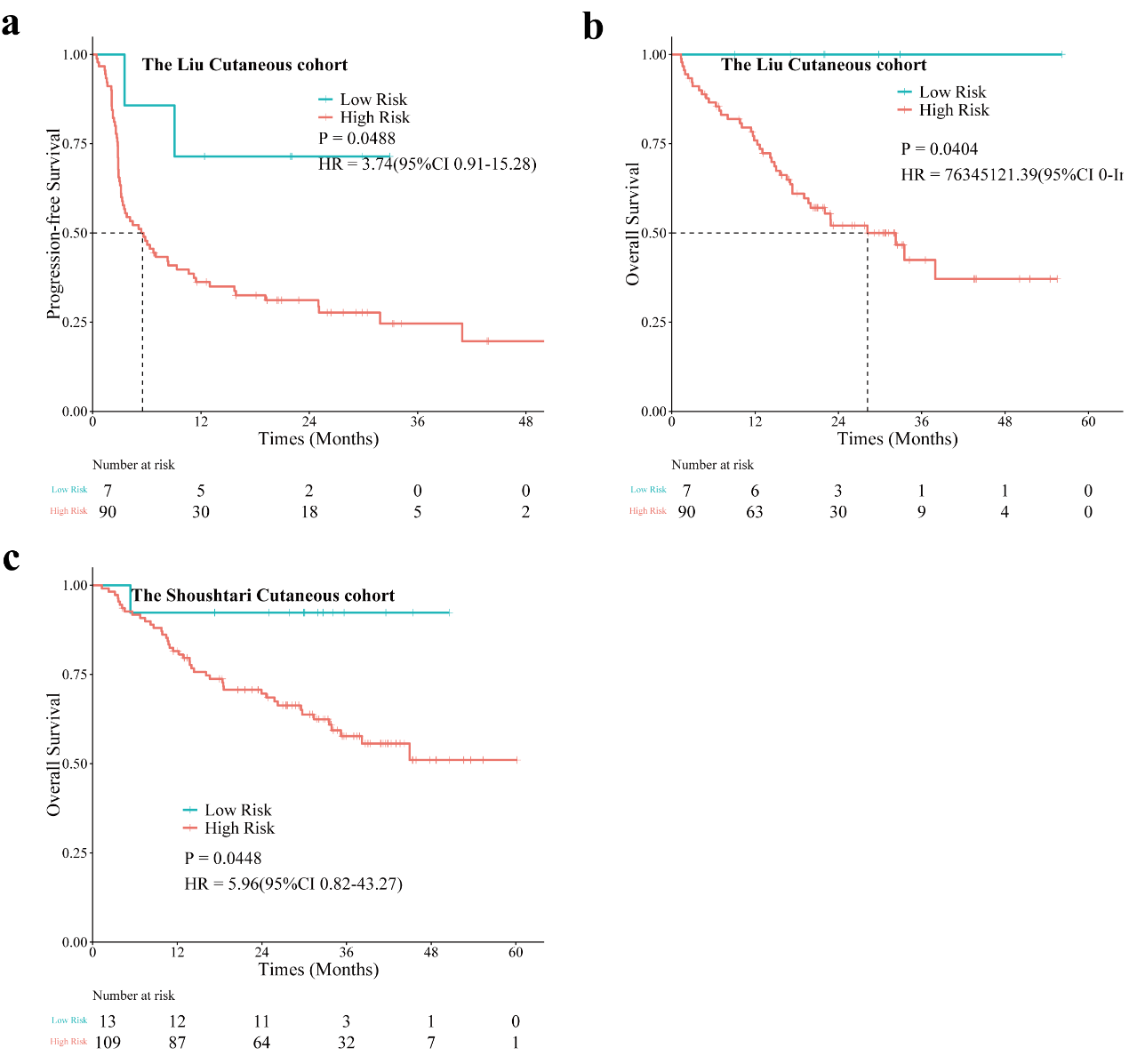


**Supplemental Figure S10.** Kaplan-Meier estimates of patients with metastatic cutaneous melanoma after anti-PD-1 treatment obtained from the second nomogram. Kaplan-Meier curves of (a) progression-free survival and (b) overall survival for patients with metastatic cutaneous melanoma in the Liu cohort. Kaplan-Meier curves of (c) overall survival for metastatic cutaneous melanoma patients in the Shoushtari cohort. HR, hazard ratio.


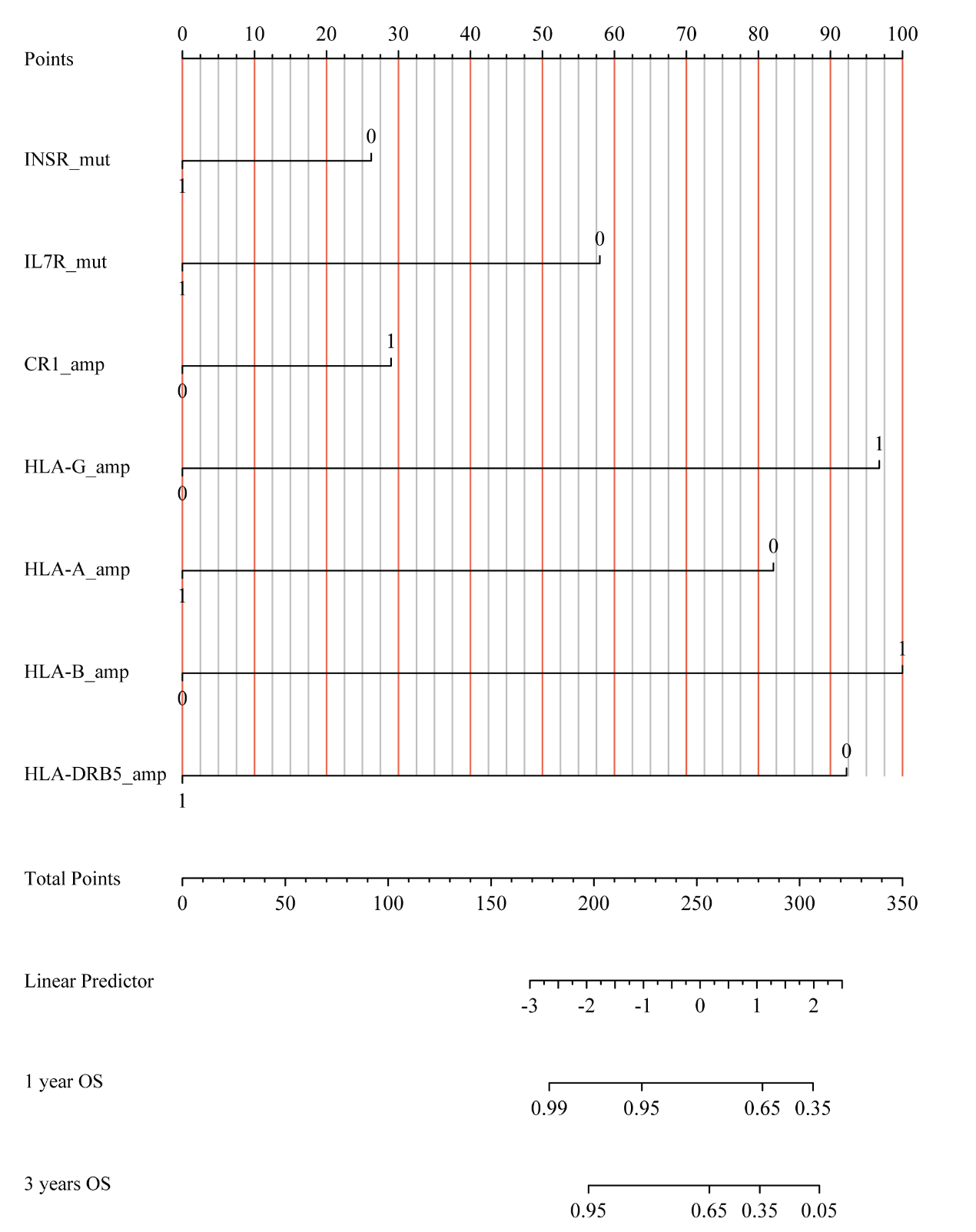


**Supplemental Figure S11.** The third nomogram using OS of the Shoushtari cohort. The number "0" and "1" designations above the lines for each gene mean wild type and mutant, respectively. "_mut" and "_amp" mean genomic mutation and CNV amplification, such as "INSR_mut" means the mutation of INSR gene and "CR1_amp" is the amplification of CR1.


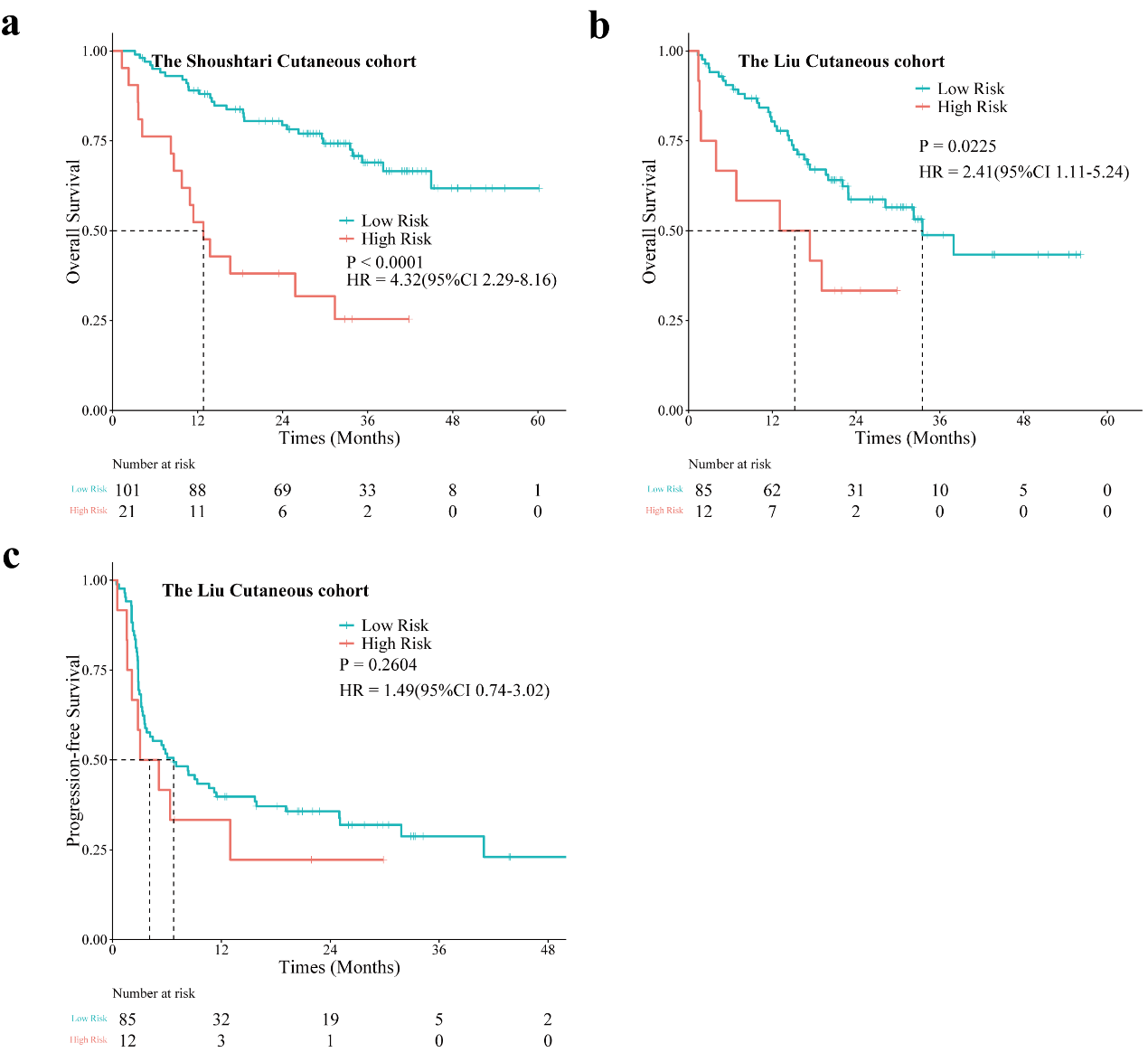


**Supplemental Figure S12.** Kaplan-Meier estimates of patients with metastatic cutaneous melanoma after anti-PD-1 treatment obtained from the third nomogram. Kaplan-Meier curves of (a) overall survival for metastatic cutaneous melanoma patients in the Shoushtari cohort. Kaplan-Meier curves of (b) overall survival and (c) progression-free survival for patients with metastatic cutaneous melanoma in the Liu cohort. HR, hazard ratio.
